# Comparison of compressive forces caused by various cannulated cancellous screws used in arthroscopic ankle arthrodesis

**DOI:** 10.1186/s13018-016-0503-x

**Published:** 2017-01-17

**Authors:** Satoshi Kamijo, Tsukasa Kumai, Shogo Tanaka, Tsuyoshi Mano, Yasuhito Tanaka

**Affiliations:** 1 Department of Orthopaedic Surgery, Fujimori Hospital, Matsumoto, Nagano Pref. 390-0811 Japan; 2 Department of Orthopaedic Surgery, Nara Medical University, Kashihara, Nara 634-8522 Japan; 3 Department of Sports Medicine, Nara Medical University, Shijo-cho 840, Kashihara, Nara 634-8522 Japan; 4 Industrial Research Institute of Shizuoka Prefecture, Aoi-ku, Shizuoka City, 421-1298 Japan

**Keywords:** Ankle arthrodesis, Cannulated cancellous screw, Compressive force, Talocrural joint, Miniature pressure sensor

## Abstract

**Background:**

When performing arthroscopic ankle arthrodesis for end-stage ankle arthritis, internal fixation is performed using bone screws after appropriate preparation.

However, optimal characteristics of bone screws have not been examined in terms of pressure force. Objective comparisons of bone-screw performance may provide information on procedures for arthroscopic ankle arthrodesis. The study objectives were to determine whether it was possible to measure compressive force changes using the newly developed device and to infer all screw characteristics from measurement results when used in actual surgeries. In addition, we performed experiments on cadavers to verify whether the experimental results could be applied to the joints of living subjects.

**Methods:**

Three types of screws (S1, S2, and S3) were inserted into the unique measurement device, and the changes in pressure were measured for each 45° turn. Changes in pressure and maximum pressure force were recorded after the application of the screws. After reaching the maximum pressure in the simulated bone, further screw rotations were accompanied by a gradual pressure decrease to 0 MPa. We also measured pressure changes in a similar manner by inserting a miniature pressure sensor into the talocrural joints of cadavers.

**Results:**

The mean maximum pressure ± standard deviation for S1, S2, and S3 were 0.832 ± 0.164 MPa, 0.434 ± 0.116 MPa, and 0.414 ± 0.127 MPa, respectively. Pressure slopes to the maximum did not significantly differ between the screws in the simulated bone, and a subsequent pressure decrease to 0 MPa was significantly more rapid for S1 than for S2 and S3. Although pressure failure after the overtightening of screws was only observed in the simulated bone, patterns of pressure vs. rotation angle were similar in simulated and cadaveric bones. The pressure profile characteristics of three different screw types were determined.

**Conclusions:**

We were able to measure the compressive force changes using the newly developed device when the screws were inserted. On the basis of the measurement results, we were able to infer the characteristics of all screws when used in actual surgery.

## Background

Ankle arthrodesis is presently considered the gold standard treatment for end-stage ankle arthritis, although at least 40 different operative procedures have been employed in the treatment of this condition [1]. The ankle arthroscopic approach was first described in the 1980s and is now widely used as an effective and minimally invasive treatment [2, 3]. In a review by Abicht and Roukis [4], the average rate of nonunion in arthroscopic ankle arthrodesis is 8.6%, which is higher than those in other joint arthrodesis procedures. However, this is not a satisfactory result. For example, the nonunion rate of 6.5% in finger distal interphalangeal joint arthrodesis or 2.0% in hallux metatarsophalangeal joint arthrodesis [5, 6] is lower than that in arthroscopic ankle arthrodesis. The issue regarding how to further decrease the nonunion rate in cases of arthroscopic ankle arthrodesis remains unresolved.

Although nonunion has been attributed to surgeon skill levels with the arthroscopic and postoperative protocol [2, 7], these factors have not been investigated for internal fixation surgery.

When performing arthroscopic ankle arthrodesis after appropriate preparation, two to four screws are placed within the range of the cancellous bone of the talus while maintaining the alignment of the talocrural joint for initial fixation. The strength of the initial fixation is an important factor that determines the final outcome. Therefore, to securely position the screw in an appropriate location within the limited available space of the cancellous bone of the talus with almost no adjustment, the surgeon must be very careful. However, practitioners continue to subjectively evaluate methods for using screws. Nevertheless, we believe that it is important for surgeons to use screws on the basis of the objective evaluation of screw characteristics.

Currently, screws are objectively evaluated using the pull-out test [8, 9], and maximum pressure is often assessed using a pressure-sensitive film [10, 11]. However, these methods do not directly evaluate the pressure force and do not enable the evaluation of pressure changes. No detailed examinations of inter-talocrural joint pressure force caused by screws have been conducted to date.

Here, we developed a new measurement device and investigated whether it is useful for measuring pressure changes. We specifically measured the pressure changes using cannulated cancellous screws with a device attached, a device with which we have had experience in arthroscopic ankle arthrodesis by inserting the screws into the device, thereby simulating insertion during actual surgery. In addition, on the basis of our measurements, we identified the methods of using screws during actual surgery that were effective. Finally, to verify that these experimental results were applicable to talocrural joints of living beings, we conducted pressure measurements on fresh human cadaveric talocrural joints (hereafter referred to as “cadaver joint”).

## Methods

### Measurement device

**Fig. 1:**
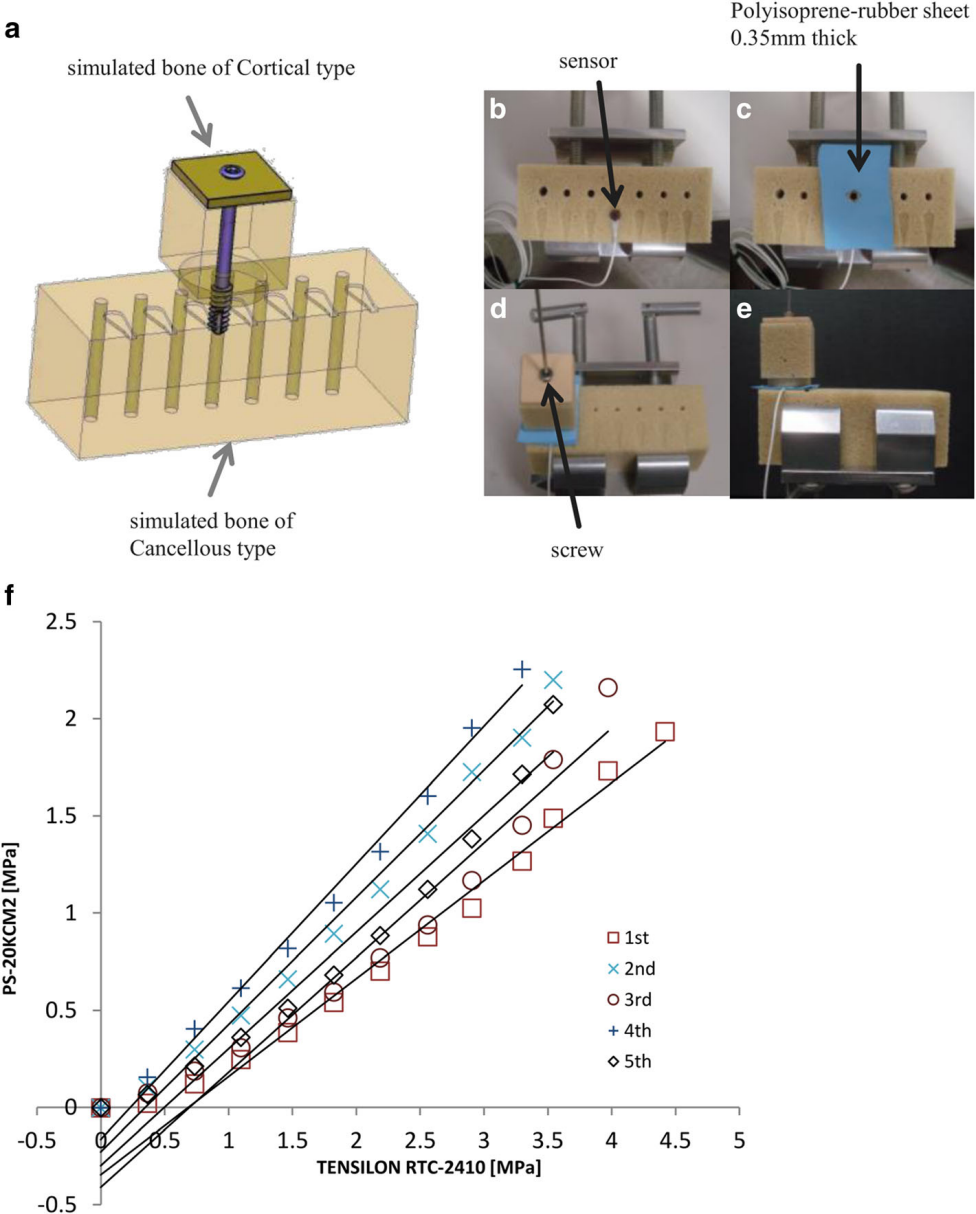
Machining of the simulated bone and procedure for the assembly of the apparatus. Schema of the apparatus: pilot holes are drilled prior to inserting screws (**a**), overhead view of sensor installation (**b**), placement of the polyisoprene rubber sheet on the sensor (**c**), rotation of the screw into the hole of the apparatus (**d**), and side view of the entire apparatus (**e**). Verification of the output characteristics of the miniature pressure sensor (**f**)

Measurements were conducted using a miniature pressure sensor (PS-20KCM2, which has a diaphragm-type strain gauge, with a sampling rate of 0.02 s (50 Hz), sensitivity of maximum 2 MPa, and rated output of 0.910 mV/V ± 1%, Kyowa Electronic Instruments Co., Ltd., Tokyo, Japan). Changes in pressure were transferred to a personal computer (PC) via an interface (PCD-300A; Kyowa Electronic Instruments Co., Ltd., Tokyo, Japan) and were then graphically visualized in real time using PCD-30A software (Kyowa Electronic Instruments Co., Ltd., Tokyo, Japan). Numerical data were recorded on a PC. To set up the sensor in a precise horizontal position at the same level as the pressure surface of the simulated bone (Sawbone®; Copyright© 2013 Pacific Research Laboratories, Inc., Vashon Island, WA), a groove was created using a machining center (Nexus 510; Yamazaki Mazak Corporation, Aichi Pref., Japan) and pilot holes were drilled prior to vertically inserting the screws in the upper surface of the simulated bone. The sensor was then installed in the groove and was wedged in place using the simulated bone. Upon the assembly of this device for screw insertion, a 0.35-mm-thick polyisoprene rubber sheet was placed on the device to ensure equal distribution of pressure to the sensor (Fig. 1a–e). To simulate surgery, cortical- and cancellous-type bones were used and a universal material testing instrument (Tensilon RTC-2410; A&D Co., Ltd., Tokyo, Japan) was used five times to monitor changes in sensor pressure with respect to the existing pressurizations. Finally, linear approximations were confirmed according to the output characteristics of the incorporated pressure sensor (Fig. 1f).

### Pressure measurements in simulated bone

**Fig. 2:**
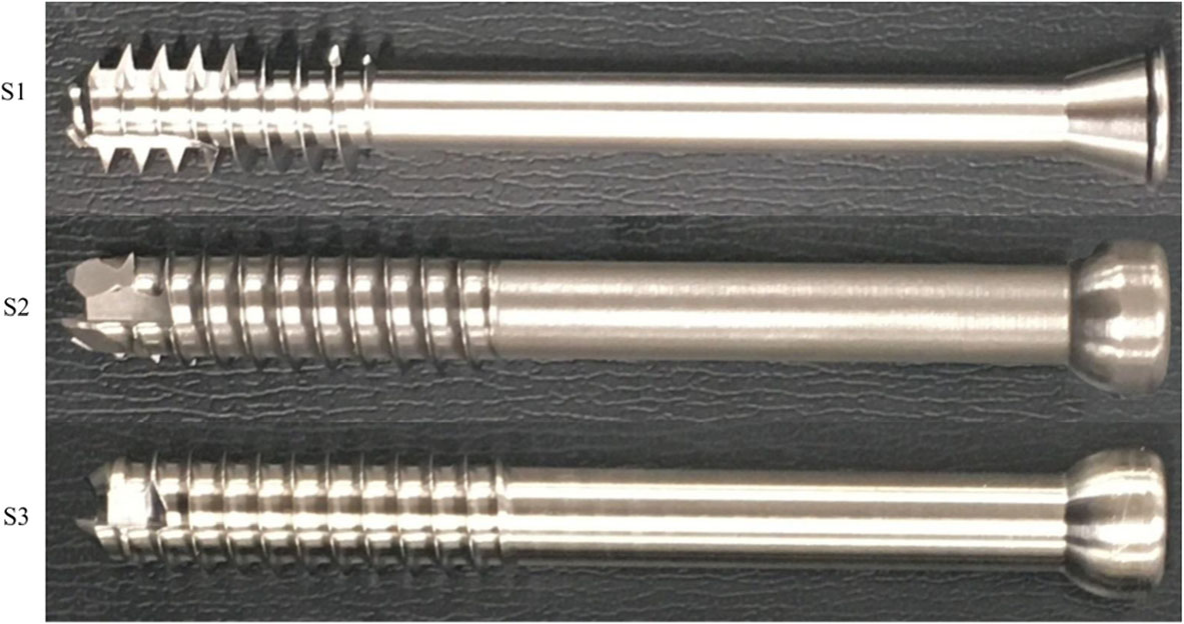
Cannulated cancellous screws S1, S2, and S3

Experiments were performed using three types of cannulated cancellous screws (S1, S2, and S3), which vary in head shape and thread length, are commonly employed in clinical practice, and have been used by us in clinical practice (Fig. 2, Table 1).

**Table 1:** Cannulated cancellous screws S1, S2, and S3

	Total length (mm)	Outer diameter	Thread (mm)	Minor diameter	Pitch (mm)
S1	55	φ 6.5	16	φ 4.0	1.75
S2	55	φ 6.5	20	φ 5.0	1.80
S3	55	φ 6.5	22	φ 5.0	1.75

Changes in pressure were measured for each 45° rotation after the insertion of S1 into the measurement device. The simulated bones were exchanged for each screw inserted, and the experiment was performed thrice with new screw holes. In all experiments, we confirmed that all pitches of the screw threads passed the interface.

Changes in pressure were graphed, and maximum pressure force and rotation angle were correlated. Subsequent measurements using S2 and S3 were performed using the same procedure.

### Pressure measurements in cadaveric joint

A fresh frozen cadaveric ankle specimen (68-year-old female) was obtained from a tissue bank (MedCure Inc., Portland, OR). The specimen lacked evidence of degenerative disease or ligamentous injury and was separated from the leg approximately 20 cm above the ankle joint, frozen at −20 °C, and thawed at room temperature before testing.

**Fig. 3:**
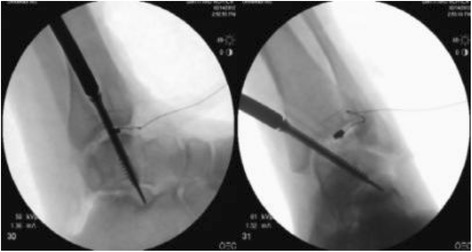
The skin was initially incised at the minimum range in front of the ankle joint, and screw pressures were then measured after the sandwiching of the waterproofed sensor in the talocrural joint

In accordance with live arthroscopic ankle arthrodesis, systematic removal and debridement of the articular cartilage as well as the subchondral bone of the cadaveric joint was initially performed using arthroscopy. Subsequently, the smallest required incision was made, while maintaining the anatomical structure of the ankle, except the joint surface at the front of the ankle, to expose the talocrural joint. Thereafter, a miniature pressure sensor was encapsulated in rubber for waterproofing and was wedged into the talocrural joint (Fig. 3). Using normal operative procedures, S1, S2, and S3 were inserted from the medial side of the tibia, i.e., the proximal site of the medial malleolus, and were then rotated in eight increments of approximately 45° per turn for each pressure measurement. Measurements for each screw were conducted using the same cadaveric joint, although the insertion site was changed for each screw. As in the simulated bone, changes in pressure force were transferred to a PC using an interface and were graphically visualized in real time using PCD-30A software. All numerical data were recorded on a PC.

### Statistical analyses

Ekuseru-Toukei 2012 software (Social Survey Research Information Co., Ltd., Tokyo, Japan) was used for statistical analyses. Differences between screws were identified using one-way analysis of variance (ANOVA) and Tukey–Kramer’s multicomparison test. Significant correlations were identified using ANOVA, and differences between regression coefficients were identified using Student’s *t* test. All tests were two-tailed. These data are presented as adjusted *R*-square and *P* values; however, data other than these are expressed as mean and standard deviation (SD); *P* values of <0.05 were considered to be statistically significant.

## Results

### Pressure force of screws in simulated bone

**Fig. 4:**
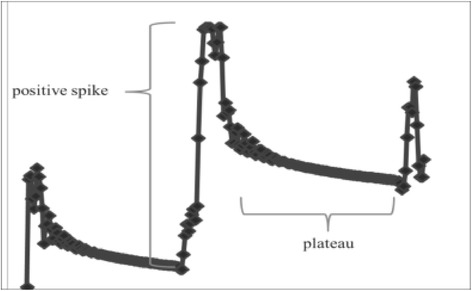
All graphs show similar step-like shapes, with positive spikes followed by a gradual pressure decrease and plateau. Positive spikes occur during the rotation of the screw, followed by a plateau

**Fig. 5:**
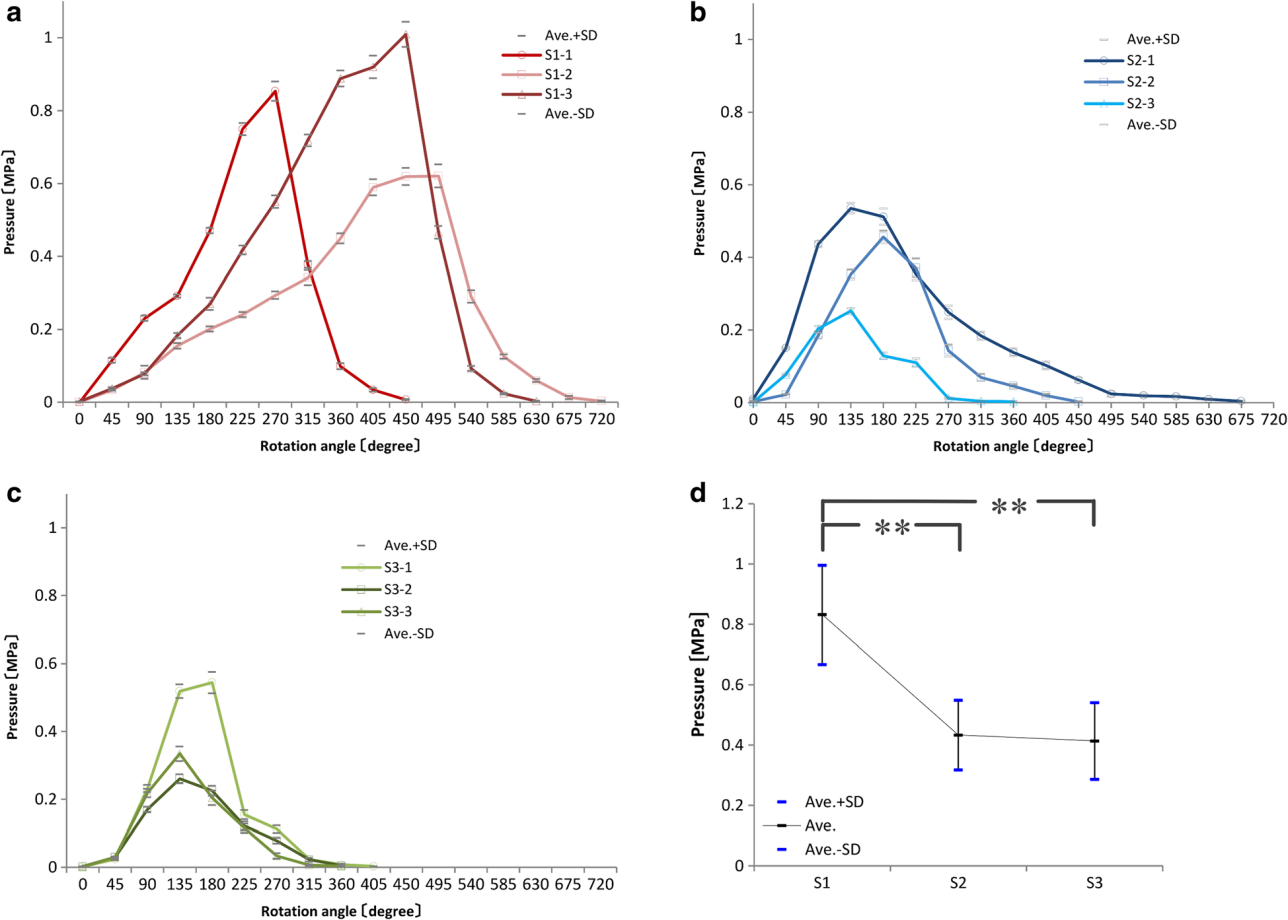
Relationships between pressure and rotation angle. The horizontal axis indicates the sum of 45° rotation increments, and the vertical axis shows the pressure (**a**–**c**). Assessment of differences in the mean maximum pressures using the Tukey–Kramer multiple comparison test (**d**)

Data were plotted on a graph of time (s; horizontal axis) and pressure force (MPa; vertical axis), and all graphs showed similar step-like patterns, with positive spikes for S1, S2, and S3 at each time interval from the beginning of the pressure force measurement and then gradual decreases and plateaus thereafter. Real-time visual confirmation on the PC monitor showed that pressure spikes occurred during rotation and then decreased and plateaued for each 45° increment (Fig. 4). It was assumed that the pressure spikes reflected the force that was exerted by the surgeon’s arm and was transmitted via the inserted screw on the simulated bone. Moreover, despite the effects of elastic force on the simulated bones, a gradual decrease in plateau pressure suggested that the pressure force was primarily due to the screw. Therefore, a gradual decrease and plateau were graphed in triplicates for S1, S2, and S3 (S1-1 to S3-3), and the pressure force of each plateau section was expressed as the mean maximum pressure force (vertical axis) against the rotation angle (45° increments; horizontal axis; Fig. 5a–c). All graphs indicating an increase in pressure force from the beginning of each increment in rotation angle and a subsequent decrease to 0 after the maximum pressure was achieved were correlated with a further increment in rotation angle.

The mean maximum pressures for S1, S2, and S3 were 0.832, 0.434, and 0.414 MPa, respectively, and were significantly greater for S1 than for S2 and S3 (*P* < 0.01). No significant difference in maximum pressure was observed between S2 and S3 (*P* = 0.27; Fig. 5d, Table 2).

**Table 2:** Assessment of the differences in the mean maximum pressures using the Tukey–Kramer multiple comparison test

	Average (MPa)		*P* values
S1 vs. S2	0.832	0.434	<0.01 (**)
S1 vs. S3	0.832	0.414	<0.01 (**)
S2 vs. S3	0.434	0.414	0.27

**Fig. 6:**
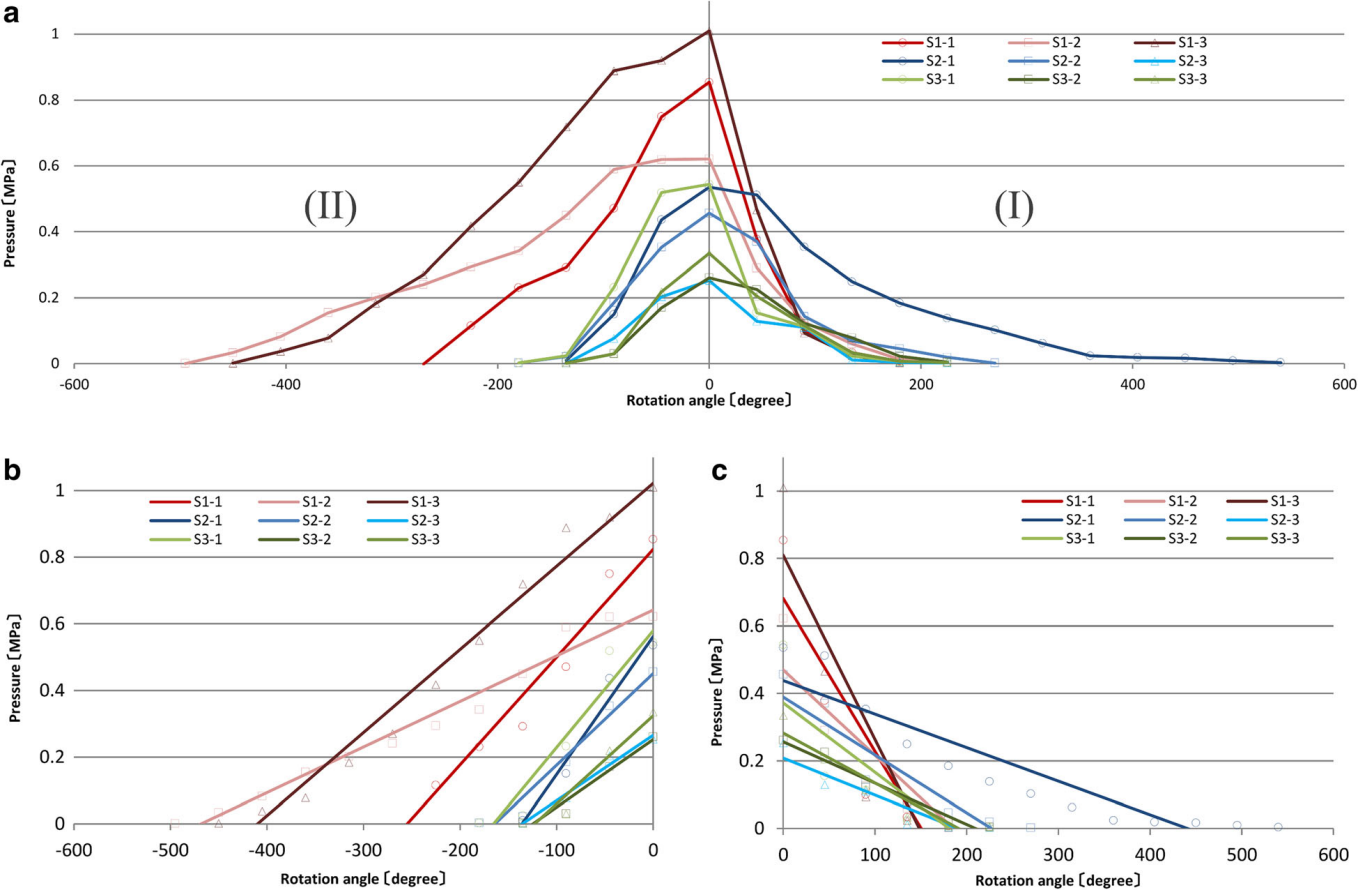
The second quadrant shows pressure increases (*II*), and the first quadrant shows subsequent decreases from maximum pressure (*I*) for all screw types in triplicate (**a**). Regression lines for pressure increases (**b**). Regression lines for pressure decreases (**c**)

**Fig. 7:**
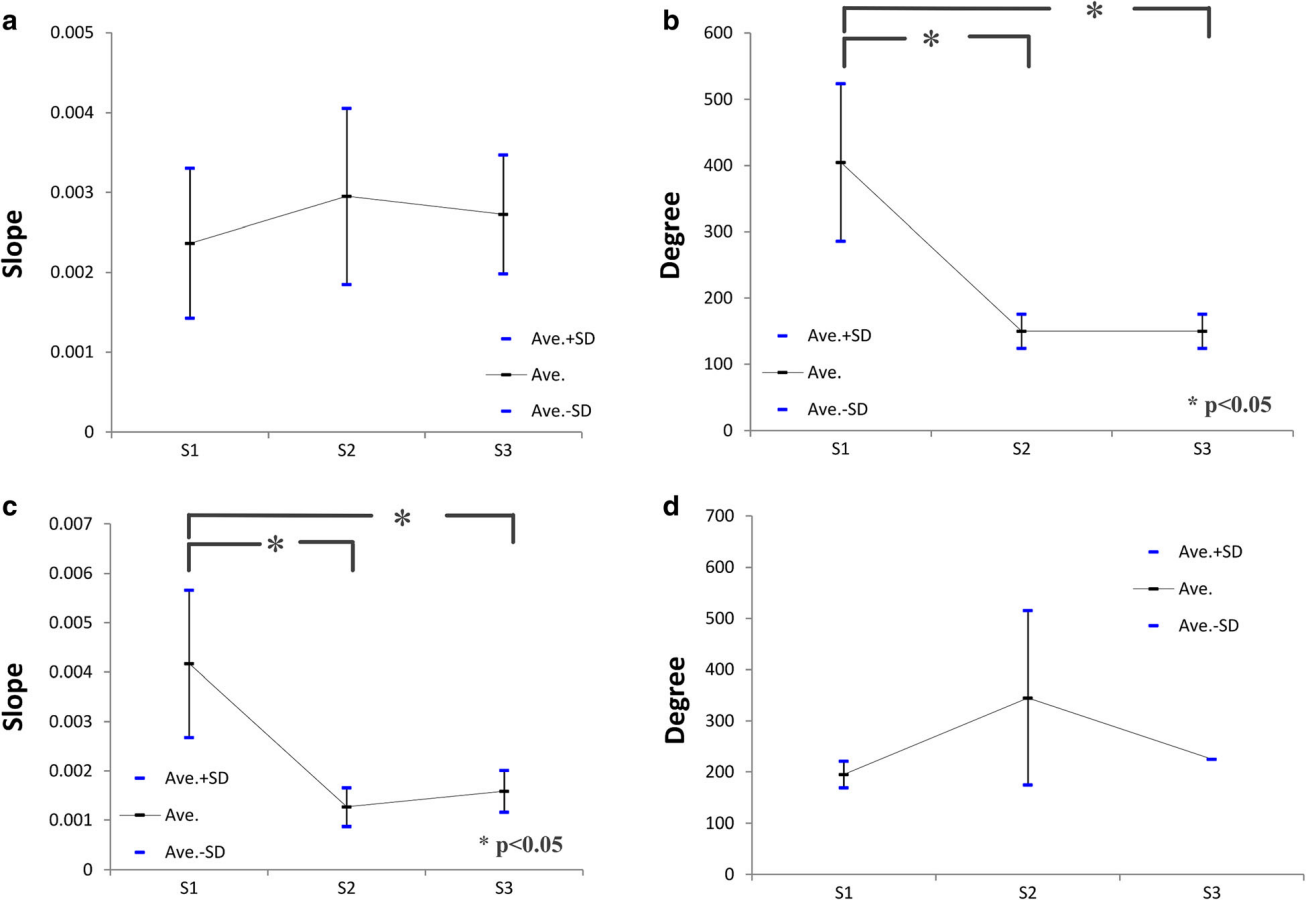
No significant differences were observed between the mean regression coefficients of S1–S3 in the second quadrant (**a**). A significant difference was observed between the average angle of S1 and S2 and that of S1 and S3 in the second quadrant (**b**). A significant difference was observed between the mean regression coefficients of S1 and S2 and S1 and S3 in the first quadrant (**c**). No significant differences were observed between the average rotation angles of S1 to S3 in the first quadrant (**d**)

In subsequent analyses, the rotation angle was plotted against the plateau pressure for S1 to S3 in triplicates to show the slopes of increasing and decreasing pressure around the maximum pressure (Fig. 6a). Significant regression coefficients were observed from the start to the maximum pressure in each graph (Fig. 6b), with adjusted *R*-square values of >0.8 (Table 3). The mean pressure slopes for S1, S2, and S3 were 0.00237 ± 0.000939, 0.00295 ± 0.00110, and 0.00273 ± 0.000742, respectively, and the associated regression coefficients did not significantly differ (*P* = 0.75; Fig. 7a). In contrast, the mean angles ± SD until the maximum pressure was achieved for S1, S2, and S3 were 405° ± 119°, 150° ± 26°, and 150° ± 26°, respectively, and were significantly greater for S1 than for S2 and S3 (*P* < 0.05). No significant differences were observed between S2 and S3 (*P* = 1.00; Fig. 7b). Subsequently, the regression analyses of angles at maximum pressure were summarized for each screw (Fig. 6c); these analyses indicated significant correlations for all screws (adjusted *R*-square > 0.6; Table 3). The mean slopes for S1, S2, and S3 were 0.00417 ± 0.00149, 0.00127 ± 0.000391, and 0.00159 ± 0.000426, respectively, and were significantly greater for S1 than for S2 and S3 (*P* < 0.05). No significant differences were observed between S2 and S3 (*P* = 0.91; Fig. 7c). In contrast, the mean rotation angles at a pressure of 0 for S1, S2, and S3 were 195° ± 26°, 345° ± 170°, and 225° ± 0°, respectively, and did not differ significantly (*P* = 0.23; Fig. 7d).

**Table 3:** Adjusted *R*-square values and *P* values of regression coefficients (Fig. 6b, c, respectively)

	Adjusted *R*-square value	*P* value of regression coefficient
In the second quadrant		
S1-1, 2, 3	0.958, 0.972, 0.973	<0.001, <0.001, <0.001
S2-1, 2, 3	0.950, 0.946, 0.963	<0.05, <0.01, <0.05
S3-1, 2, 3	0.895, 0.921, 0.909	<0.01, <0.05, <0.05
In the first quadrant		
S1-1, 2, 3	0.760, 0.753, 0.758	<0.05, <0.05, <0.05
S2-1, 2, 3	0.835, 0.810, 0.824	<0.001, <0.01, <0.01
S3-1, 2, 3	0.623, 0.953, 0.864	<0.05, <0.001, <0.01

### Pressure force of screws in cadaveric joint

**Fig. 8:**
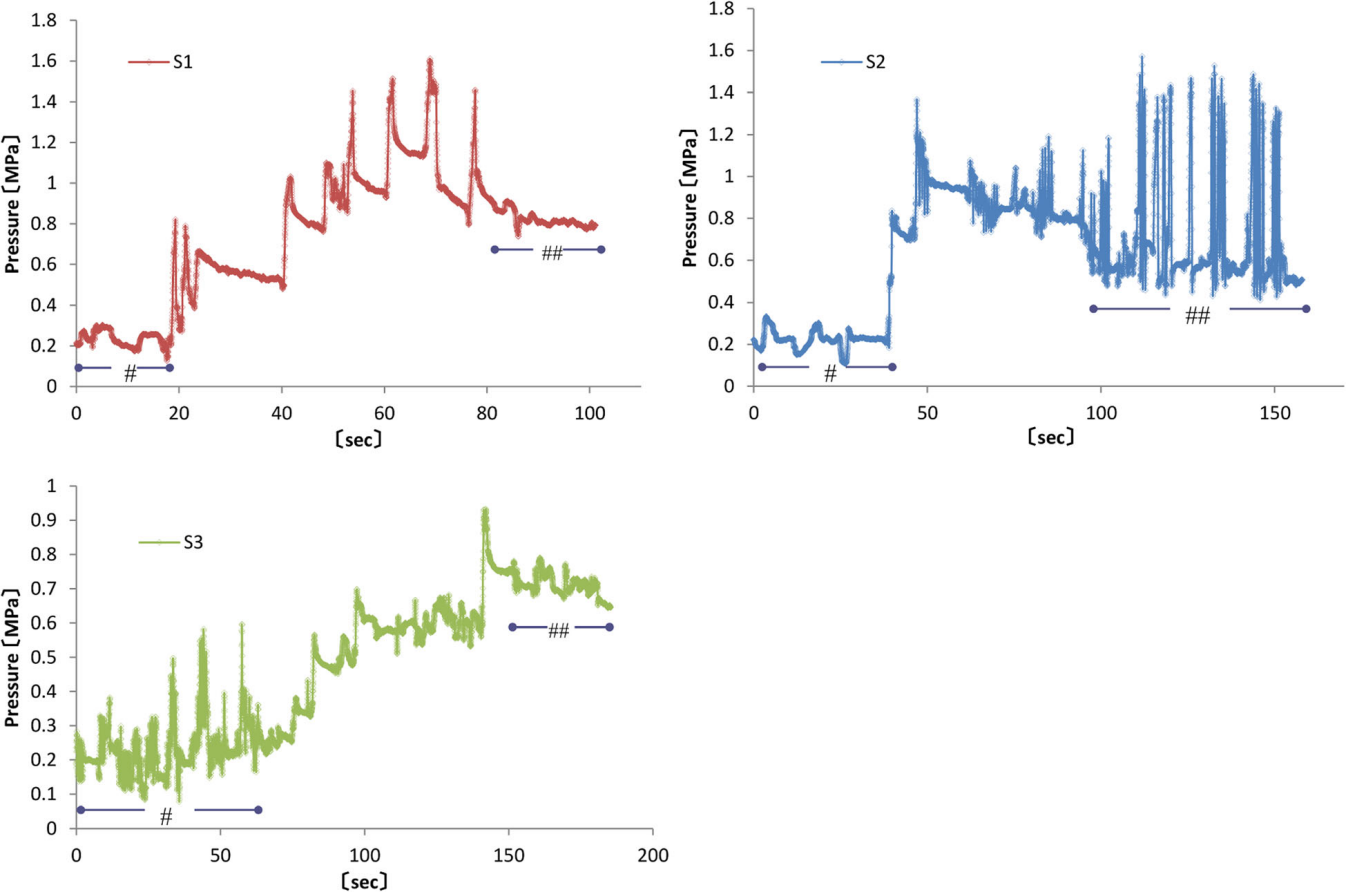
Screw pressures in the cadaveric bone. All graphs show similar step-like shapes, with positive spikes followed by a gradual pressure decrease and plateau. Initial pressure was greater than 0 MPa (#). After achievement of maximum pressure, the screw torque was reduced but the pressure did not immediately decrease (##)

Plots of time (s; horizontal axis) against pressure force (MPa; vertical axis) for each screw showed step-like patterns, with positive spikes for each rotation from the beginning of the pressure force measurement and a subsequent gradual decrease and plateau. Although this pattern was similar to that observed in the simulated bone (Fig. 8), a constant pressure force was detected from the beginning of the measurement (“#” in Fig. 8). Furthermore, because screw torque decreased during the second half of the procedure, the pressure in the simulated bone rapidly reached 0 MPa, whereas that in the cadaveric joint remained constant and higher than the initial pressure (“##” in Fig. 8).

## Discussion

In this study, we were able to confirm the characteristics of all screws using a newly developed device that utilizes a miniature pressure sensor. Initial experiments in the simulated bone accordingly indicated that S1 had a significantly greater maximum pressure than the other screws. Among the present screws, S1 had the shortest thread length, which facilitated insertion into the limited area of the cancellous bone of the talus. Furthermore, compared with the other screws, S1 had significantly greater angles upon reaching maximum pressure, allowing ample rotation to reach the maximum pressure during surgery and reducing the likelihood of excessive screw rotation. However, the chances of insufficient screw rotation are more with S1 screws, and a higher rotation angle requires more time to increase screw torque.

In further analyses, the slope of the regression line after maximum pressure was significantly greater with S1 than with S2 and S3, indicating that when the rotation angle exceeds that associated with maximum pressure, the compressive force drops more suddenly with S1 than with the other screws. We believe that on exceeding maximum pressure, the decrease in compressive force was because of shear fracturing of the internal threads caused by excessive rotation of the screw. Although S1 produced significantly greater maximum pressure than S2 and S3 on exceeding maximum pressure, which may be because of the screw head shape that is specific to S1, compared with other screws, internal thread shear fracture occurred more rapidly, thereby rapidly decreasing the pressure. Therefore, in comparison with S2 and S3 screws, extra caution should be exercised to avoid excessive rotation of S1. In contrast, the compressive force of S2 and S3 on overtightening relatively slowly decreased, and although the maximum compressive force was inferior, we believe that the internal thread shear fractures were not likely to occur suddenly or rapidly. Therefore, we assumed that S2 and S3 can be used to apply constant compressive forces without paying much attention to the excessive rotation of screws, which is necessary when using S1, and S1 can be used to apply maximum pressure.

Therefore, based on this simulation, we anticipate that it is possible to ascertain the characteristics of screws before they are used in actual surgery.

Furthermore, except for the fact that in cadaveric joint experiments, the constant pressure force while starting measurements did not reach 0 MPa in the cadaveric joints after reducing screw torque, graphs of the cadaveric joint pressure were similar to those observed for the simulated bone. We believe that these differences arose because of periarticular soft tissues, such as ligaments, intra-articular surface tension, and the diagonally inserted screw. Therefore, even in the simulated bone, we believe that we could simulate, to an extent, the actual intra-articular pressure changes.

Limitations of this study include the assessment of only three types of screws and performance with only one cadaveric joint. Therefore, the simulated bone may require more accurate preparation using a more high-precision machining center and more controlled screwing technique. In addition, further experiments using separate cadaveric joints for each screw are required, albeit considering the differences between the specimens.

The present data warrant further comparative studies of bone screws using the procedure described here. Screw characteristics such as thread length, pitch interval, material, and presence or absence of a washer could be assessed to provide information on screw insertion procedures for arthroscopic ankle arthrodesis.

## Conclusions

The new measurement device we developed was useful for measuring pressure changes. In addition, on the basis of our measurements, we were able to confirm the characteristics of all screws and were able to analyze which methods of using screws during actual surgery were effective.

## Abbreviations

ANOVA: One-way analysis of variance
PC: Personal computer
SD: Standard deviation
